# Current Perspective on Nasal Delivery Systems for Chronic Rhinosinusitis

**DOI:** 10.3390/pharmaceutics13020246

**Published:** 2021-02-10

**Authors:** Junhu Tai, Kijeong Lee, Tae Hoon Kim

**Affiliations:** Department of Otorhinolaryngology-Head & Neck Surgery, College of Medicine, Korea University, Seoul 02841, Korea; junhu69@korea.ac.kr (J.T.); peppermint_1111@hotmail.com (K.L.)

**Keywords:** topical treatment, drug delivery, chronic rhinosinusitis, fluid dynamics, upper respiratory diseases

## Abstract

Chronic rhinosinusitis is an upper respiratory disease during which topical drug treatment via the nasal cavity is the most actively utilized therapeutic strategy. In addition to steroids, antibiotics, and antifungal agents, which are widely used in clinical practice, research on novel topical agents to improve the bacterial biofilm or mucociliary clearance remains ongoing. Moreover, owing to the complex structure of the nasal cavity, the effects of nasal drug delivery vary depending on factors related to delivery fluid dynamics, including device, volume, and compounds. In this article, we review methods and compounds that have been applied to chronic rhinosinusitis management and introduce recent advances and future perspectives in nasal drug delivery for upper respiratory diseases.

## 1. Introduction

Chronic sinusitis (CRS) is a common disease with global prevalence rates of 10.9% in Europe [1], 13% in the United States [2], 6.95% in South Korea [3], and 8% in China [4]. CRS is a chronic inflammatory nasal disease with a course of over 12 weeks and is diagnosed as CRS with or without polyps according to the presence of nasal polyps (NPs) [5]. Several pathogenic factors have been attributed to the development of CRS, including the presence of biofilms, changes of mucociliary clearance, and remodeling of tissue [6]. To eradicate biofilms and increase mucociliary clearance, local medication is very effective; therefore, treatment with local therapeutic agents has been increasingly considered as an important type of CRS treatment. Numerous new compounds and drugs have been developed for CRS. Saline and corticosteroids remain the most important in the local treatment of CRS; however, charged or hydrophilic drugs are unable to adequately cross the biofilm [7]. Moreover, owing to the rapid mucociliary clearance, the residence time of drugs in the cavity is markedly short [8], which may seriously limit the passive diffusion of drugs through the epithelium. Nasal administration is a promising way of drug delivery [9] but necessitates a good device for improved drug delivery. A nasal spray is the most commonly used nasal drug delivery equipment, presenting advantages of portability and convenience. However, it also has some disadvantages; e.g., the drug may fail to reach the entirety of the sinuses and superior nasal parts, is discharged into the throat by nasal cilia, swallowed into the stomach, or cannot play a role in the treatment of nasal diseases. Moreover, patients may experience an unpleasant taste, odor, or feel on using nasal sprays [10]. The problems associated with most nasal drug delivery devices include the particle size of drops or powders, the location and form of drug deposition, and the loss of drugs from the nasal cavity after administration. To resolve these problems, various nasal drug delivery devices with new functions have been developed. This review categorizes the types and characteristics of delivery methods and drugs developed to date and introduces the newly developed devices.

## 2. Compounds

### 2.1. Saline

Saline nasal irrigation (SNI) is known to be useful for patients affected by CRS. SNI is a safe way of treatment in the CRS. SNI is usually performed with saline or other solutions and improves the mucosal function of the nasal cavity owing to direct mucosal cleansing [11]. In addition, SNI enhances ciliary beat frequency by increasing sol layer hydration and propelling gel layer. Chong et al. [12] reviewed that large volume irrigation with 150 mL of a saline solution (hypertonic) was better than a placebo, and the group with large volume SNI showed mild symptoms of nasal congestion, sinus headache, and frontal pain than the control group that only allocated to standard therapy. Succar et al. [13] revealed that the most common method of administration is delivering through a low-pressure, high-volume device. The factors influencing the composition of nasal saline include sodium chloride tonicity, oligo-elements, minerals, and temperature [13]. Hypertonic solution is defined as a solution with more than 0.9% sodium chloride, while hypotonic solution is that with under 0.9% sodium chloride. In addition, seawater, which contains natural minerals and oligo-elements, is also used for nasal irrigation. A meta-analysis comparing hypertonic saline irrigation with isotonic saline irrigation reported that patients with sinusitis benefited more with improved symptoms from hypertonic saline irrigation than from isotonic saline irrigation, especially in the younger population [14]. Another meta-analytic study recently claimed that hypertonic saline irrigation is more effective in treating CRS; however, there was no difference in smell improvement compared to isotonic saline irrigation [15]. In a double-blind randomized controlled trial for the clinical effects of various nasal irrigation formulations, nasal irrigation using lactated Ringer’s solution showed a better effect on sinonasal symptom improvement than either normal saline or hypertonic saline solution [16]. As for nasal irrigation using seawater, hypertonic seawater was reported to reduce CRS symptoms more than isotonic seawater did [17]. Furthermore, an in vitro study comparing non-diluted seawater and normal saline using airway epithelial cells suggested that non-diluted seawater improved ciliary beat frequency and wound repair speed [18]. Recently, clinical studies using hypertonic seawater irrigation identified that hypertonic seawater was effective for reducing the symptom score and endoscopic score in aspirin-induced CRS as well as CRS with nasal polyps [19,20].

### 2.2. Corticosteroids

Corticosteroids, the most potent anti-inflammatory agents, are often used to control CRS [21]. There is considerable evidence that topical corticosteroids are often used in the treatment of patients with CRS. A study [22] reported that large-volume corticosteroid irrigation improves the symptoms of patients with CRS after sinus surgery. The author highlighted that corticosteroid irrigation should be considered as a part of important therapy in postsurgical CRS. Some studies have evaluated the adverse events of nasal corticosteroids [23,24,25,26]. These studies have observed that nasal corticosteroids are safe. No major adverse events happened. Intranasal corticosteroids used for chronic rhinosinusitis are listed in Table 1. The first-generation corticosteroids include beclomethasone dipropionate, flunisolide, budesonide, and triamcinolone. The second-generation consisted of fluticasone furoate, fluticasone propionate, ciclesonide, mometasone furoate (MF), and betamethasone sodium phosphate. The most widely used corticosteroids administered via intranasal spray are fluticasone propionate, MF, and beclomethasone. From a recent Cochrane review of intranasal corticosteroid use for CRS, studies comparing intranasal use of fluticasone propionate and beclomomethasone dipropionate in CRS patients reported no difference in overall symptom improvement between both groups [27]. In addition, no difference in the improvement of sinonasal symptoms was observed between intranasal fluticasone propionate and MF. Triamcinolone is usually applied via nasal dressing material following endoscopic sinus surgery. Prospective clinical studies comparing triamcinolone versus normal saline-impregnated nasal dressing reported that greater reduction in edema, crusting, scarring, and olfactory function improvement was identified in the triamcinolone-soaked nasal packing group than in the normal saline-soaked packing group [28,29]. Budesonide, applied through saline irrigation, was found to reduce symptom score and endoscopic appearance score in CRS patients, especially those with eosinophilia, when 1 mg of budesonide was administered daily [30]. Another study also reported a significant improvement in symptoms for eosinophilic CRS patients treated with budesonide rinse group compared to those in the normal saline irrigation group [31]. In contrast, Thamboo et al. [32] identified no symptom score improvement in the budesonide rinse group, whereas budesonide applied via mucosal atomization showed a significant effect on symptom reduction.

### 2.3. Antibiotics

Multidrug-resistant bacteria and polymicrobial biofilms are still a major challenge [33]. Topical antibiotic agents are a research hotspot recently because they can provide higher concentrations of antibiotics locally and limited systemic absorption [34]. A previous study compared nebulized antibiotics to nebulized saline; although CRS symptoms were found to be improved, nebulized antibiotics failed to offer additional benefits when compared with saline [35]. According to the recent studies, it is currently not recommended to use nebulized antibiotics for patients with CRS, but nebulized antibiotics seem to improve the quality of life, especially in terms of social function and pain in some patients with CRS with practically no side effects. In the future, culture-oriented nebulized antibiotic therapy may be an option for patients who do not respond to conventional therapy [36]. Typical antibiotics used topically for CRS are listed in Table 1. A meta-analysis of 6 studies about the use of mupirocin saline irrigation (440–500 mg/L saline) in patients with recalcitrant staphylococcal CRS indicated that short-term use of topical mupirocin was effective in reducing residual staphylococcal infection [37]. For *Pseudomonas aeruginosa* cultured-CRS patients, inhalation of tobramycin affected the reduction of pathogen colonization [38]. In addition, it was found that the decrease of bacterial biofilm and the recovery of normal airway epithelium and cilia function were also affected by the application of 0.3% ofloxacin eye drops to the middle meatal mucosal specimens for 12 weeks [39]. A retrospective study of 58 patients with recalcitrant CRS treated with high-volume topical antibiotics via irrigation based on nasal culture results (vancomycin, levofloxacin, mupirocin, gentamicin, ceftriaxone, tobramycin, and ceftazidime) demonstrated an improvement in symptom score and endoscopic appearance [40]. A recent prospective study also showed the superiority of topical antibiotics (vancomycin, mupirocin, tobramycin) combined with topical steroids in methicillin-resistant *Staphylococcus aureus* eradication compared to topical steroids alone [39,41]. However, because the evidence level of the previous studies was low, The European Position Paper on Rhinosinusitis and Nasal Polyps 2020 (EPOS2020) disproved the efficacy of topical antibiotics [5,30,31,32].

### 2.4. Antifungals

Antifungal therapy remains controversial in CRS treatment. Regarding the topical antifungal treatment of CRS, amphotericin B can be used for nasal spray or nasal irrigation. A study has revealed that no significant difference in computed tomography scores was seen between the topical antifungals and the control group [42]. Another recent study reported that amphotericin B irrigation in CRS with nasal polyps significantly improved CT score compared to normal saline irrigation, however, was shown to have no effect on recurrence rate [43]. Khalil et al. [44] suggested that topical application of fluconazole, either via irrigation or a nasal spray, could reduce the recurrence rate of allergic fungal rhinosinusitis compared to a conventional medical treatment group or oral antifungal treatment group. A Cochrane review has evaluated the effects of topical amphotericin B as well as fluconazole in CRS; however, owing to the low credibility of the available evidence, it cannot be determined with absolute certainty whether the use of topical antifungals has a positive role in patients with CRS [45].

### 2.5. Decongestants

Kirtsreesakul et al. [46] evaluated the effectiveness of oxymetazoline treatment with nasal steroid therapy, which is considered superior to using nasal steroid only, and no rebound congestion which develops from the overuse of nasal decongestant sprays was observed. This is consistent with results observed in allergic rhinitis, which may indicate that using nasal decongestant and nasal corticosteroid at the same time can prevent rebound swelling [47,48]. In another clinical trial, Humphreys et al. [49] compared the difference of nasal topical decongestant and xylometazoline add to a saline spray in functional endoscopic sinus surgery (FESS) during the early postoperative period; however, no difference was observed between the two groups. This indicates that when the nasal cavity is severely blocked, a decongestant can be considered to add in the nasal steroid spray. However, more clinical trials are warranted to ensure the safety and efficacy of this combination.

### 2.6. Novel Therapeutic Agents

#### 2.6.1. Surfactant

Surfactants spontaneously combine to form micelles. When used as an additive, surfactants can increase additional hydrophobicity and biodegradation, therefore, they promote mucociliary clearance [50]. Additionally, they possess immediate antibacterial functions, including the disruption of biofilms [51]. Biofilm is difficult to eradicate due to calcium ion bridges, which produce gels, which greatly enhance their physical structure in order to resist degradation [50]. Citric acid/zwitterionic surfactant (CAZS) is a new type of surfactant composed of citric acid. The citric acid can chelate calcium ions in calcium ion bridges [52] and the zwitterionic surfactants can separate the biofilm from the mucosal surface and force it to dissolve. In vitro, CAZS showed a good effect on the removal of biofilm [53], but ciliary toxicity was found in preclinical animal studies [54]. SinuSurf is a proprietary surfactant that reportedly reduces the population of several species of bacteria, but because of its toxic effect, it was withdrawn from the market [55]. More research on counteracting surfactant toxicity is needed.

#### 2.6.2. Hyaluronic Acid

Hyaluronic acid (HA) is a kind of glycosaminoglycan composed of disaccharide basic structure. It is widely distributed in connective tissue, epithelial tissue, and nerve tissue, and it can prevent the transmission of macromolecular substances and infectious media through filtration [56,57]. Its therapeutic effects on CRS with or without NPs have been documented, including its effects on mucosal repair, free radical generation, and mucociliary clearance. Hyaluronic acid is well known for its benefits to the upper respiratory tract, such as prevention of bronchoconstriction induced by inflammatory mediators and reduction of human neutrophil elastase [58]. It shows good anti-adhesion and antibiofilm effects in vitro, especially for *Staphylococcus aureus*. Its presence in bacterial biofilm (BBF) is related to the severe clinical situation of CRS. Some studies have reported the anti-BBF effects of HA in vivo, especially in the form of atomized sodium hyaluronate plus normal saline [59]. Cassandro et al. [60] divided 80 patients with CRS and nasal polyposis without FESS into 4 groups. They were treated with normal saline, hormone spray, HA, and hormone spray plus HA, respectively. Analysis of symptoms, radiologic reports, nasal manometry, and saccharin clearance tests showed improvement in all steroid and/or HA treated groups, and the combination of HA and corticosteroid was more effective.

#### 2.6.3. Colloidal Silver

Because of the emergence of antibiotic resistance, new alternative therapies are needed. Colloidal silver (CS) shows anti-biofilm properties in multidrug-resistant bacteria [61]. The suspension of submicroscopic silver particles does not directly attack bacteria but leads to the inactivation of enzymes responsible for bacterial respiration, reproduction, and metabolism by forming homo-base pairs with guanine, targeting sulfhydryl group to form S-silver bonds, and mediating membrane alteration [62]. Ooi et al. [63] used a mixture of water, sodium citrate, silver nitrate, and potassium iodide to prepare CS and treated patients with CRS through CS nasal irrigation. Although the Sino-Nasal Outcome Test (SNOT-22) scores and endoscopic scores were improved, they were not better than those of the antibiotic treatment group. A study involving 22 patients evaluated the safety and efficacy of CS as a local treatment in patients with refractory CRS with NPs and observed no meaningful subjective or objective improvements [64].

#### 2.6.4. Xylitol

Xylitol is a natural antibacterial agent and was first isolated from the bark of beech trees in 1890, it can inhibit the growth of bacteria by destroying glucose cell wall transport and intracellular glycolysis [65,66]. Lysozyme, lactoferrin, and β defensins in the airway surface constitute a part of the local defense system. They have stronger antibacterial activity at low salt concentrations, and xylitol is an osmolyte that has a low transepithelial permeability which can reduce the salt concentration so to improve the ability of nasal mucosa airway surface to kill respiratory pathogens [67,68]. Jain et al. [69] investigated the effects of xylitol on the biofilms and growth of bacteria and compared them with the control group. This study revealed that 5% and 10% xylitol got a good effect on anti-biofilms.

#### 2.6.5. Manuka Honey

It has been found that Manuka honey (MH) has a good anti-biofilm effect on gram-positive and gram-negative bacteria [70,71]. Methylglyoxal in Manuka honey not only inhibits bacterial growth but also has immunomodulatory effects, which can promote wound healing and tissue regeneration [72]. However, it is difficult to reach a consensus on its use owing to the lack of consistent efficacy data, Lee et al. [73] found no significant difference between MH sinus irrigation group and saline sinus irrigation group in patients with CRS. The experimental results reported by Ooi et al. [74] revealed that 6 of 10 (60%) patients sinonasal rinses MH demonstrated a reduced bacterial culture rate, with no major adverse events.

## 3. Methods of Nasal Drug Delivery

**Figure 1:**
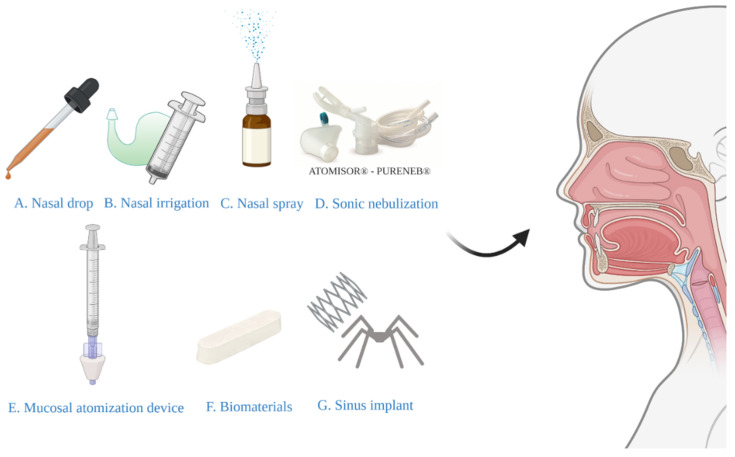
Various types of devices for nasal drug delivery systems.

In the treatment of CRS, traditional devices for nasal local administration include methods such as nasal drops, nasal irrigation, and nasal sprays [75]. In recent years, sonic nebulization, mucosal atomization devices, biomaterials, and sinus implants have been developed. This article will introduce their characteristics and advantages, as well as disadvantages (Figure 1).

### 3.1. Nasal Drops

Nasal drops (Figure 1A) have historically been the simplest and most convenient system for nasal drug delivery [76]. The disadvantage of this system is the lack of dose accuracy, so nasal drops may not be suitable for prescription products. However, although drops are effective for some people, their popularity is limited by the need for a head-down position and/or extreme neck extension required for gravity-driven drop deposition [77]. The Kaiteki position is an effective way to deliver drops to the olfactory epithelium. Lying on the side with the head tilted down 20–30° and turn the chin-up 20–40° and drop the medicine into the upper nostril. Aim at the upper edge of the nasal septum mucosa and hold it for 30 s [78]. A systematic review has recommended the positions of lying head back and lateral head low [79]. The author stated that the effect of the two methods was equivalent and superior to that of head back and head down positions. Glucocorticoid solutions of fluticasone propionate and betamethasone are commercially available as nasal drops in the United Kingdom and Europe [80]. A study including 54 patients with CRS [81] demonstrated the efficacy of fluticasone propionate nasal drops, revealing a significantly greater improvement in symptoms, nasal airflow, and polyp volume than placebo groups.

### 3.2. Nasal Irrigation

**Figure 2:**
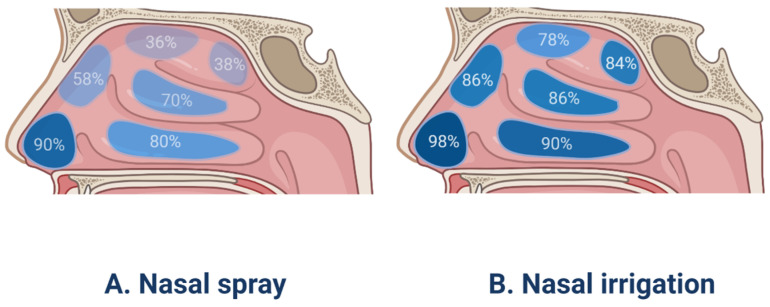
The surface area coverage of drugs using nasal spray and nasal irrigation in the different parts of the human nasal cavity.

Nasal irrigation (Figure 1B), also termed nasal wash, rinse, douche, or lavage, can reduce the severity of infections of the nasal cavity and sinuses. Furthermore, it is typically recommended for patients after sinus surgery [82]. Syringes, pots, and various types of squeeze bottles can be employed for nasal irrigation and can improve mucociliary clearance, as well as symptoms of nasal stuffiness and obstruction [83]. A previous study has evaluated nasal irrigation systems in terms of their physical rinsing parameters [84], for the whole nasal cavity and paranasal sinuses flushing, it is recommended that the compressible flushing system has a better minimum output pressure of 120 mbar, a good connection between the outlet and the nostril (possibly inserted into the nasal vestibule), and an upward flushing flow (45°). However, low-volume drug delivery is not as effective as high-volume drug delivery in penetrating paranasal sinuses [85]. In the international consensus statement of allergy and rhinology: rhinosinusitis in 2016, it is strongly recommended that large volume (>200 mL) nasal saline irrigation be used as an auxiliary means for other drug treatment of CRS [86]. A recent study involving 418 patients with rhinosinusitis showed that large-volume nasal irrigators were more effective than other types of irrigators in removing nasal secretions and reducing postnasal drip [87]. Kanjanawasee et al. [88] compared the effects of nasal irrigation using hypertonic saline (HS) and isotonic saline (IS) in treating sinonasal diseases. The results showed that HS improved symptoms over IS in treating sinonasal diseases; however, HS presented an increased number of minor side effects than IS. However, it remains controversial which saline demonstrates greater clinical effects in patients with CRS. A study has compared the effect of Dead Sea Salt (DSS) irrigation and DSS nasal spray with saline irrigation and local nasal steroid spray. The results showed that both groups showed significant improvement in mean Sino-Nasal Outcome Test 20 (SNOT-20) scores following treatment, but the degree of improvement did not significantly differ between the two groups [89]. Kent et al. compare the distribution of nasal irrigation to nasal spray by scoring the cadaveric specimen surface area stained by methylene blue [90], and no significant difference was observed in the nasal vestibule, inferior turbinate, and middle turbinate between nasal irrigation and nasal spray; however, nasal irrigation was distributed more widely than nasal spray in the sphenoethmoidal recess, superior turbinate and ostiomeatal complex (Figure 2). Harvey et al. [23] gave 2 mg mometasone to 44 patients with CRS by nasal spray or nasal irrigation in the treatment of CRS after sinus surgery and showed that a one-year posttreatment blockage, drainage, fever, and total visual analog scores were all lower in the corticosteroid irrigation group than nasal spray group.

### 3.3. Nasal Spray

A nasal spray is a simple device with a piston; on pressing the pump head, the piston sucks water into the lower pump column connected to the plastic pipe and the piston, and the water is introduced into the spray head to form a mist and spray out. This is a very rapid process that transforms the liquid into droplet phase within 100 µs after leaving the nozzle, accelerating the flow velocity from 0 to 15–20 m/s [91]. The deposition and penetration of drugs from nasal spray to nose are mainly determined by liquid atomization in the spraying nozzle and aerosol formation, and the released drug droplets are mainly deposited by impact, and then due to the interaction of airflow, the deposited liquid drug diffuses along the nasal surface. [92]. Nasal spray (Figure 1C) is very suitable for the long-term daily administration of drugs; solution and suspension can be prepared into nasal spray [93]. Owing to the availability of metering pumps and actuators, the nasal spray can provide an accurate dose of 25 to 200 μL. The choice of pump and actuator components depends on the particle size of the drug and the viscosity of the preparation [94].

As shown in Figure 2, it has been identified that the drugs administered using nasal spray devices, which produce larger particles (10–150 μm) at high speeds, are deposited in the anterior portion of nasal cavity, rather than in the main nasal passage [95]. Various efforts have been made to facilitate the deposition of drugs administered using nasal spray into the main nasal passage by controlling the droplet size, spray angle, viscosity, and breathing patterns. Cheng et al. [96] discovered that more droplets are deposited in the main nasal passages when the droplets are smaller and that the spray plume angles are narrow. Another study conducted using a silicone nose model reported that formulations with lower viscosity showed greater distribution in nasal passage when sprayed than those with higher viscosity [97]. However, according to the same study, the breathing pattern did not affect the aerosol distribution. Furthermore, Foo et al. [98] proposed both a narrow plume angle (<30°) and administration angle (30°) as the most important factors for enhancing deposition efficiency on the main nasal passage and suggested that droplet size, inspiratory flow rate, and viscosity are relatively minor factors. Recently, the same group reported differences in spray plume angle to enhance the deposition efficiency between adults and children; plume angles less than 40° and 20° (most narrow) showed the most improved deposition of drugs in adults and 12-year-old children, respectively [99]. In addition, a study on particle deposition via nasal spray using 15 particle sizes and 3 breathing patterns reported that larger volume median diameter increases particle deposition in the anterior nasal cavity, whereas smaller volume diameter reduces anterior regional distribution and induces deposition in the main nasal passages under sniffing inhalation or constant inhalation conditions [100].

Topical nasal steroid sprays have been shown to demonstrate minimal systemic absorption and can be safely used as long-term maintenance therapy in patients with CRS [101]. The analysis of data showed that the use of topical steroid sprays is good for symptoms, recurrence of polyps, polyp size, and the airflow in the nasal cavity [102]. All types of nasal spray products will provide correct directions for use in patient information leaflets. However, a doctor or pharmacist should demonstrate how to properly use the nasal spray device [103]. To improve drug efficacy and reduce the possibility of side effects, patients should be informed of the accurate method of using nasal spray devices. Ganesh et al. [104] surveyed how patients use intranasal steroid sprays in 103 patients, revealing 20 patients with epistaxis, with 80% using an ipsilateral hand technique which uses the same hand to the same nostril. Patients with nasal steroid spray using ipsilateral hands are more prone to nosebleed than those who use the contralateral spray technique.

### 3.4. Sonic Nebulization

In 1959, Guillerm et al. [105] demonstrated that aerosols can diffuse through the sinuses by increasing sound, that is to say, the circulation of air and the penetration of aerosol into nasal sinuses can be increased by the resonance of air and sinus orifice. Sonic nebulization (Figure 1D) uses a 100 Hz sound with a jet nebulizer generating the aerosol. Herein, a breath-enhanced nasal jet nebulizer improves drug administration during patient inspiration and reduces drug leakage into ambient air during exhalation. Durand et al. reported the sonic nebulization optimized aerosol deposition in the nasal cavities and effectively targeted anatomic regions of interest [106]. Sonic nebulization enhances the penetration of aerosols into the maxillary sinus using the acoustic hyper-pressure in the ostium, with 3–5 times greater deposition in the paranasal sinuses than nebulizers without sonic boost [107,108]. Reychler et al. [109] reported that compared with nasal spray, the volume of aerosol inhalation of budesonide in the nasal cavity was lower, but farther, which supports highly atomized drugs better than aerosol spray to reach the olfactory area. These results can explain the difference in olfactory function between sonic aerosol inhalation and nasal spray during the same dose of corticosteroid administration.

### 3.5. Mucosal Atomization Device (MAD)

The MAD (Figure 1E) consists of an atomizing nose nozzle head connected to a standard 1 cc or 3 cc syringe. It can atomize in any position and the malleable stylet allows 180° positioning of the nasal plug. High pressure applied to the plunger ensures MAD to transform liquid medicine into a 30–100 μm fine mist and effectively deliver the medicine into the nasal cavity [110]. Several studies have suggested that nasal aerosol inhalation is a more effective local drug delivery method than nasal spray because it produces small and slow particles that pass through the nasal cavity and cover the larger surface of nasal mucosa [111]. Clinical studies have shown that local application of budesonide through MAD can reduce the demand for systemic prednisone and improve the overall evaluation scores of doctors and patients after CRS [112]. Although short-term use of nebulized topical nasal steroids for less than 2 months has been reported to be safe and effective, Manji et al. [113] suggested that long-term topical budesonide nasal administration through MAD is at risk of adrenal suppression and elevated intraocular pressure. Moffa et al. [114] compared the following nasal devices: nasal syringe-irrigation, nasal spray, MAD, and some other devices, using a color-based method in human corpse models to determine which device is more effective in delivering topical medication. The results revealed that compared with traditional sprays, MAD nasal spray provided a more effective way to deliver local drugs to deeper and higher parts of the nasal cavity. Furthermore, cadaver specimens with lying-head-back position during drug administration using MAD showed an increased distribution of the drug to paranasal sinuses including frontal, ethmoid, and sphenoid sinus compared to those with head-down and forward position, indicating head position could be a critical factor for drug delivery using MAD, especially for patients with refractory CRS [115].

### 3.6. Biomaterials

For several years, biomaterials (Figure 1F) for CRS have been used in postoperative settings. Its basic principle is stopping bleeding, prevention for adhesion, improving patency of ostium-opening, and local drug administration. Commonly used biomaterials include polylactide sinus implants, polyurethane foam, and carboxymethylcellulose [116]. One of the FDA-approved biomaterials is Sinu-Foam™, which is a carboxymethyl cellulose polysaccharide material. When it is hydrated, it forms gelatin, which is used to place the ethmoid sinus cavity after endoscopic sinus surgery (ESS). However, in one study, placing Sinu-Foam^TM^ in the middle nasal cavity had no effect on improving the outcomes of endoscopic procedures [117]. With the continuous development of CRS postoperative management technology, many materials have emerged to provide effective local corticosteroids into the postoperative sinuses, including SinuBand^®^, NasoPore^®^, and Merocel^®^ [118]. Sinuband^®^ bioabsorbable implant is a 2 cm × 2 cm film with mucinous and nonadhesive surfaces, and its matrix is fibrinogen. Sinuband^®^ contained 160 μg fluticasone propionate, which was released over time after implantation. Gwijde et al. [119] reported that Sinuband^®^ was superior to Merocel in polyp scores. David et al. [120] used a 4 cm dressing(NasoPore^®^) impregnated with 2 mL of 40 mg/mL triamcinolone acetonide solution for patients with CRS. The results showed that the use of absorbable nasal packing containing triamcinolone acetonide can significantly improve early postoperative healing. A recent study compared non-absorbable Merocel^®^ packs with steroid-eluting absorbable stents [121], revealing that patients with Merocel^®^ packs achieved improvements in their SNOT-22 scores at postoperative visits.

### 3.7. Sinus Implants

The entry of local steroids to the nasal cavity and sinuses may be obstructed by different factors, and hence, novel modes of drug delivery into sinonasal cavities need to be studied. Steroid-eluting sinus implants (Figure 1G) have been introduced as a new method to optimize surgical outcomes and to treat recurrent nasal polyposis after ESS by delivering locally sustained-release corticosteroids directly to inflammatory sinus tissues [122]. This can create an effective sinus drug delivery system, local use of corticosteroids to solve the problem of inflammation [123]. Currently used FDA-approved steroid-eluting sinus implants are Propel family products (Propel^®^, Propel Mini^®^, Propel Contour^®^) and SINUVA™. The implants have made a significant contribution in reducing postoperative interventions and providing effective management after surgery [124]. Propel^®^ is composed of a bioabsorbable polylactide-co-glycolide polymer coated with 370 μg of the corticosteroid MF. Once implanted, the implant expands itself to fit different sizes and shapes of the ostium. Polyethylene glycol is an anti-inflammatory and anti-protein barrier that affects water retention and promotes tissue biocompatibility. It also helps to control the rate of MF elution from implants, which is determined by the diffusion mechanism regulated by drug concentration, chemical composition, matrix type, polymer morphology, and coating thickness. These factors allow the corticosteroids to spread to the surrounding mucosa in a controlled manner within approximately 30 days [125,126,127]. The second-generation MF-eluting sinus implant can give a high dose of topical steroid for about 3 months, significantly improving the postoperative symptoms [128]. The SINUVA™, like the PROPEL^®^ family, is also made up of bioabsorbable polymers, it can expand to adapt to space after surgery, and it has self-expansion and will fade in the operating cavity. More than the Propel family products, it contains 1350 μg MF and controls the slow release of drugs in about 90 days [129,130]. Long-term results showed that that the steroid-eluting implant is a kind of durable, effective, and safe treatment of CRS [131].

## 4. Recent Advances and Future Prospects

**Figure 3:**
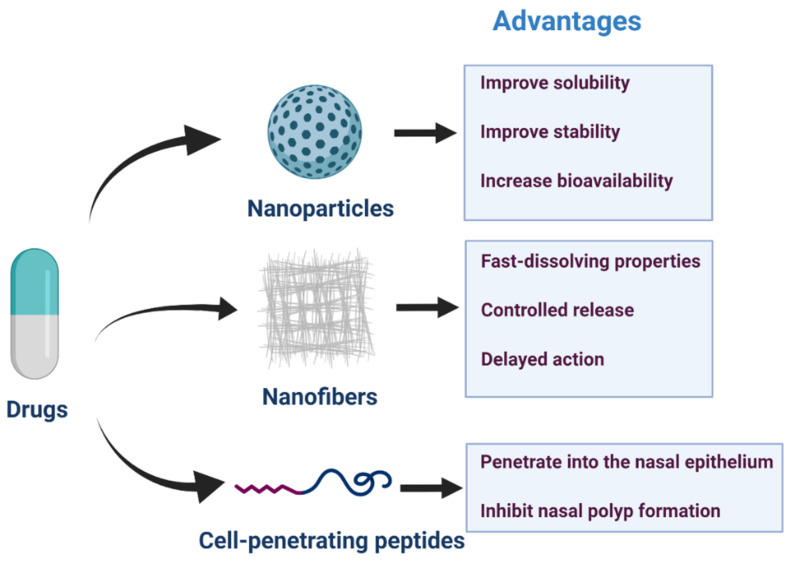
Nanoparticles, nanofibers, and cell-penetrating peptides for nasal drug delivery and advantages in drug transportation.

Recent advanced technologies, including nanoparticles, nanofibers, and cell-penetrating peptides (Figure 3), have been developed to improve drug solubility, stability, and controlled release [132,133,134]. Several studies have reported their gradually improved functions. They improved on some of the shortcomings of previous drug delivery systems.

### 4.1. Nanoparticles

IUPAC defined a nanoparticle as “a particle of any shape with dimensions in the 1 × 10^−9^ and 1 × 10^−7^ m range” in 2012 [135]. A nanoparticle is a kind of submicron particle dispersion or solid particles, which can deliver a variety of important therapeutic drugs, such as nucleic acids, peptides, and small hydrophobic and hydrophilic molecules to various biological systems. Furthermore, nanoparticles can be designed in different shapes and sizes, and their surfaces can be modified to fulfill their biological needs [136]. These nanoparticles have a common core/coating structure. The core can be inorganic or organic, and the coating is usually formed by natural polymers, synthetic biopolymers, or their combination. The coating confers water dispersibility, prevents aggregation, reduce non-specific adsorption in biological systems, and provides a platform for conjugation of targeted ligands or other functional molecules (such as chelating agents). The length, charge, hydrophobicity, and flexibility of the coated molecules, as well as the overall size, shape, and elastic modulus of nanoparticles are the key factors affecting the in vitro and in vivo properties of nanoparticles [137]. In recent years, several types of nanotherapeutic drugs have been evaluated and designed, including liposomes, polymer nanoparticles, and micelles, as carrier materials [138]. Common synthetic polymeric nanoparticles used for drug delivery include polyacrylamide [139], polyacrylate [140], and natural product-chitosan [141]. In the upper respiratory tract, there are microfold cells in the nasal passage-associated lymphoid tissue that transport antigens through the mucosa [142,143]. Microfold cells are more likely to transport smaller particles [144], and the smaller size of nanoparticles is the preferred size for absorption by microfold cells [145]. A previous review concluded that the process of nanoparticles entering microfold cells is related to caveolin-1, clathrin, micropinocytosis, and toll-like receptor mediated-stimulation [132]. Broza et al. [146] reported a cross-reaction nanoarray based on molecular modified gold nanoparticles, which was used to analyze respiratory samples to screen patients with CRS. The results showed that its specificity, sensitivity, and accuracy were all higher than 80% for patients with CRS and the control group. Compared with conventional dosage forms, nanotechnology-based drug delivery can overcome some anatomical, physiological, chemical, and clinical barriers. Nanoparticle systems can provide treatment to areas of the body that other delivery systems cannot reach [147]. The advantages of nanoparticles include improving the solubility and stability of drugs, increasing the bioavailability of the target, and prolonging the action time by controlling the release rate. This can reduce side effects and provide a more convenient method of drug delivery, so as to improve patient compliance and treatment effects. The results of Jumana et al. [148] showed that under the simulated pH condition of nasal mucosa microenvironment, the release of MF from poly lactic-co-glycolic acid (PLGA) nanoparticles in vitro showed an initial burst release, followed by a sustained release phase. The kinetics of drug release follows an anomalous non-Fickian transport, which is due to drug diffusion through the polymer, polymer erosion, swelling, and degradation. This suggests that nanoparticles can be lyophilized to obtain stable nanoparticles so as to reduce the initial burst release. It was reported that the degradation rate of PLGA increased with an increase in glycolic acid units, and the polymer with 50:50 ratio of lactic acid and glycolic acid had the fastest degradation rate [149]. *Staphylococcus aureus* infection and biofilm can affect the progression of chronic sinusitis and postoperative complications, Zhang et al. [150] developed nanoparticles that loaded isosorbide mononitrate combined with anti-*Staphylococcus aureus* α-toxin antibody to study its anti-biofilm effect. The results showed that it almost completely destroyed the structure of the biofilm, which provides a very meaningful prospect for the treatment of infectious diseases caused by biofilm. Lai et al. [151] developed mucus permeation granules composed of PLGA and Pluronics, which can rapidly penetrate the accumulated and highly viscoelastic mucus in sinuses of patients with CRS. Their findings give the support to the development of mucus-penetrating nanodrugs for the treatment of CRS. A lot of research work has been done in the field of intranasal drug delivery based on nanotechnology, and nanoparticle drugs for intranasal drug delivery are waiting to be developed [152].

### 4.2. Nanofibers

Electrospinning is a universal and simple technology that can produce nanofibers suitable for various biomedical applications by optimizing parameters for electrospinning and/or combining with other methods. Moreover, physical, biological, and chemical cues can be easily generated on electrospun nanofibers in a controllable and reproducible manner [153]. Nanofibers are fibers whose diameters are in the nanometer range, and different polymers can be used for nanofibers and so the nanofibers get different kinds of physical properties and form to different applications [154]. Many polymers have been used as matrices for the preparation of nanofibers such as poly(vinyl alcohol), poly(ethylene oxide), poly(ε-caprolactone), poly(acrylic acid), ethyl cellulose, cellulose acetate, hydroxypropylmethyl cellulose, poly(acrylonitrile), cellulose acetate phthalate, and poly(urethane) [155]. Nanofibers can enhance cell attachment, drug loading, and mass transfer properties through an inherently high surface-to-volume ratio; consequently, various drugs have been incorporated into nanofibers [156]. It is very important to understand the histology of the mucosa before preparing mucoadhesive nanofiber formulations. According to the characteristics of newly developed drugs and the physiology and anatomy of the mucosal surface, we can select the appropriate candidate drugs for transmucosal delivery stringently [157,158]. Drug delivery systems based on nanofibers have some advantages, including masking the taste of bitter drugs, a simple fabrication process, and superior pharmaceutical and pharmacokinetic performances when compared with regular delivery systems. Moreover, adhesive nanofibers have many characteristics, such as rapid dissolution, controlled release, and delayed action of drugs [159]. The drug release kinetics of nanofibers can be regulated by the selection of polymer, calixarene, cellulose, and other matrix materials, as well as the preparation process of nanofibers. For different tissue microenvironments, the pH value of the drug delivery system is different. The sustained-release behavior of the drug is mainly due to the polyelectrolyte behavior of the matrix in the nonprotonated state at acidic pH, which leads to controlled drug release through diffusion. Their physicochemical behavior in the microenvironment is very important for controlling drug release. The morphology and diameter of the nanofibers were also fine-tuned to influence the controlled drug release [160]. Youhui et al. [14] showed that intranasal self-assembled peptide nanofiber vaccines may be a new, needle-free, and adjuvant-free method to induce protective immunity against bacterial and fungal infections involving skin and mucosal barrier surfaces. Most recently, Gholizadeh et al. [161] combined a new carbon nanofiber modified carbon electrode with a human nasal epithelial mucosa on a chip to achieve real-time quantitative monitoring of nasal administration in vitro. Compared with the traditional nasal drug transport detection technology, it can save more economic expenditure and time; however, it still needs further development and validation to replace the past detection technology.

### 4.3. Cell-Penetrating Peptides (CPPs)

Hydrophilic or charged drugs demonstrate difficulty in penetrating the epithelium. Furthermore, due to the rapid clearance of mucociliary, these drugs remain in the nasal cavity for a short time, which may seriously limit their passive diffusion through the nasal epithelium. CPPs are a family of peptides, usually composed of 5–30 amino acids. These amino acids can pass through tissues and cell membranes through energy-dependent or energy independent mechanisms, and have no interaction with specific receptors [162]. Reagents such as phosphodiamidomorpholine oligomers, peptide nucleic acids, peptides, proteins, and small drug molecules can be covalently coupled with CPP through chemical bonds (such as disulfide or thioester bonds) or through cloning and subsequent expression of CPP fusion protein, the HIV-1 trans-activator of transcription(TAT) protein, penetratin, polyarginines, DPV1047, MPG (*N*-methylpurine DNA glycosylase), Pep-1, and peptide derived from vascular endothelial cadherin(pVEC) are the most structural and functional CPPs, most of them are in preclinical or clinical development stage [163]. In recent years, increasing attention has been paid to the application of CPPs in the intracellular delivery of low-permeability molecules; CPPs translocate by forming a transient membrane structure. In this model, the penetrating dimer binds to the negatively charged phospholipid, resulting in the formation of reverse micelles in the lipid bilayer, through which peptides penetrate into the plasma membrane [164]. CPPs can transport cargo molecules to the cytoplasm by inducing endocytosis or direct membrane translocation. Endocytosis is a process in which cells take up substances, and the plasma membrane folds inward to bring substances into cells. Endocytosis is involved in the internalization of CPPs, but it has been suggested that different mechanisms may occur simultaneously. The mechanism of direct transmembrane transport is simply that CPPs directly penetrate into the biofilm through an energy independent cellular process, which may involve direct electrostatic interaction with negatively charged phospholipids [165]. CPPs possess several advantages, including simple synthesis, good tissue permeability, and good compatibility with other carriers, and have been used for the various delivery systems [166,167]. It is also suggested that the dual drug delivery system combining the advantages of CPPs and nanoparticles can improve the performance, accuracy, half-life, stability, and drug loading capacity of drug delivery systems [168]. Kim et al. [169] combined resveratrol (RSV) with amphiphilic CPP (LK) rich in α-Heli leucine (L) and lysine (k) and delivered it to nasal epithelial cells via the nose to inhibit hypoxia-inducible factor-1 α induced epithelial–mesenchymal transition (EMT). In the eosinophilic CRS with NPs mouse model, rsv-lk conjugate can penetrate the nasal epithelium and effectively inhibit EMT, NP formation, epithelial destruction, and related inflammation. The required dose is 10 times lower than that of free RSV, and the administration times are 3 times less.

## 5. Conclusions

After years of development, nasal drug delivery systems have gained considerable momentum. Nasal drops, nasal sprays, and nasal irrigation have the advantages of being simple and economical, but present disadvantages, such as inaccurate dosage and difficulty in reaching the depth of the nasal cavity. In recent decades, sonic nebulization and MAD have been developed, making great progress in nasal atomization. Biomaterials and sinus implants have rendered nasal drug delivery more durable and effective post-surgery. However, improving BBF and mucociliary clearance remains an issue that has not been completely resolved. Therefore, in recent years, research on nanoparticles, nanofibers, CPPs, and other new drug delivery systems is gradually increased. Their development has positive significance for drug penetration, delivery of low-permeability molecules, controlled release, and so on, and more functions are being found and studied. Further studies and clinical applications are needed to clarify the long-term clinical efficacy better and guarantee the safety of these interventions.

## Figures and Tables

**Table 1:** Classification of topical agents generally used in chronic rhinosinusitis.

Classification	Compounds
Saline	Isotonic saline (0.9%), Hypertonic saline (>0.9%), Hypotonic saline (<0.9%) Ringer-Lactate solution Isotonic/Hypertonic seawater
Corticosteroid	1st generation: beclomethasone dipropionate, flunisolide, budesonide, triamcinolone 2nd generation: fluticasone furoate, fluticasone propionate, ciclesonide, MF, betamethasone sodium phosphate
Antibiotics	Carboxylic acid: mupirocin Glycopeptide: vancomycin Aminoglycoside: neomycin, tobramycin, gentamicin Fluoroquinolone: levofloxacin Cephalosporin: ceftazidime, ceftriaxone
Antifungals	Amphotericin B, Fluconazole
Decongestants	Oxymetazoline, Xylometazoline

## Data Availability

There is no supporting data.
